# Acitretin-Conjugated Dextran Nanoparticles Ameliorate Psoriasis-like Skin Disease at Low Dosages

**DOI:** 10.3389/fbioe.2021.816757

**Published:** 2022-01-07

**Authors:** Jiajia Lan, Yuce Li, Jingjing Wen, Yu Chen, Jing Yang, Liang Zhao, Yuting Xia, Hongyao Du, Juan Tao, Yan Li, Jintao Zhu

**Affiliations:** ^1^ Department of Dermatology and Venereology, Union Hospital, Tongji Medical College, Huazhong University of Science and Technology (HUST), Wuhan, China; ^2^ Hubei Engineering Research Center of Skin Disease Theranostics and Health, Huazhong University of Science and Technology (HUST), Wuhan, China; ^3^ Hubei Engineering Research Center for Biomaterials and Medical Protective Materials, School of Chemistry and Chemical Engineering, Huazhong University of Science and Technology (HUST), Wuhan, China; ^4^ State Key Laboratory of Materials Processing and Mold Technology, School of Chemistry and Chemical Engineering, Huazhong University of Science and Technology (HUST), Wuhan, China

**Keywords:** acitretin, nanoparticles, low dosage, stat3, psoriasis

## Abstract

Psoriasis is a common chronic inflammatory skin disease mainly characterized by keratinocyte hyperproliferation and massive infiltration of inflammatory immune cells. Acitretin (ACT), an FDA-approved first-line systemic drug for psoriasis treatment, could suppress the proliferation of keratinocytes and downregulate the expression of inflammatory cytokines by modulating signal transducer and activator of transcription (STAT) signaling pathways. However, dose-dependent side effects of ACT limit its long-term administration in the clinic. Therefore, improving the therapeutic efficacy of ACT to reduce clinical dosage will benefit the patients. Here, we develop ACT-conjugated dextran nanoparticles (ACT-Dex NPs) and evaluated the potential for psoriasis treatment. Our results indicate that ACT-Dex NPs ameliorate psoriasis-like skin disease significantly at a low dosage which does not cause side effects, while neat ACT drugs at an equivalent dosage provide much less benefit. Moreover, we demonstrate that ACT-Dex NPs suppress keratinocyte proliferation more efficiently than neat ACT by enhancing the inhibitory effect on STAT3 phosphorylation. Thus, the proposed ACT-Dex NPs provide an effective and safe option for psoriasis treatment.

## 1 Introduction

Psoriasis has affected ∼ 2% of people worldwide. The quality of life of psoriatic patients was negatively impacted due to the repetitive relapsing and remitting during their lifetime. (Parisi et al., 2013; Boehncke and Schön, 2015; Michalek et al., 2017) The characteristics of psoriasis are epidermis thickening and an inflammatory infiltrate of dermal and epidermal immune cells. The crosstalk between keratinocytes and immune cells through pro-inflammatory cytokines leads to feedforward amplification of psoriasis inflammation. (Lowes et al., 2014)

Acitretin (ACT), as the active metabolic product of etretinate, has been developed as a first-line systemic drug for treating moderate-to-severe psoriasis. (Carretero et al., 2013; Nast et al., 2015; Chiricozzi et al., 2017) It could modulate the signaling pathways related to the signal transducer and activator of transcription (STAT), thus suppressing the keratinocyte proliferation and downregulating the inflammatory cytokine expression). (Niu et al., 2012; An et al., 2017; Qin et al., 2017) Clinically, ACT could benefit patients with psoriasis at proper doses, which should not be lower than 25 mg/day. (Goldfarb et al., 1988; Gupta et al., 1989; Katz et al., 1999; Chularojanamontri et al., 2019) However, clinicians face a dilemma when setting the doses because both the therapeutic effects and side effects of ACT are dose-dependent; *i.e.*, lower doses result in less efficacy, while higher doses result in significant side effects. These side effects include mucocutaneous effects (e.g., dry skin, skin peeling, cheilitis, alopecia, or rhinitis), hyperlipidemia, hepatotoxicity, ophthalmologic effects, and teratogenicity. Thus, long-term administration of ACT is limited in the clinic. (Katz et al., 1999; Lee and Koo, 2005) A few endeavors have been targeted toward improving the therapeutic efficacy of ACT at lower doses. For example, the combination of low-dose ACT and methotrexate remitted psoriasis-like skin lesions more effectively than monotherapy. (An et al., 2017) However, combined therapies may increase the frequency of adverse effects, and the administration is complicated which could reduce patient compliance. Several studies have developed ACT-loaded nanogel through nanostructured lipid carriers (NLCs), star-shaped polymethacrylic conjugates, chitin-based systems, or niosome-based systems for topical ACT delivery. (Agrawal et al., 2010; Divya et al., 2016; Abu et al., 2018; Mielanczyk et al., 2020) Unfortunately, the development of topical delivery systems may not be the optimum of ACT modification considering the indication of ACT that involves systemic organs in severe psoriasis. (Heath et al., 2018) Therefore, there is an urgent demand for novel delivery systems of ACT with better therapeutic efficacy and safety.

Nanomaterials have attracted much interest for biomedical applications due to their chemical and size-dependent physical properties. (Chen et al., 2021; Dutta et al., 2021) Nanoparticle (NP)-based drug delivery systems could improve the therapeutic efficacy of various drugs due to multiple advantages, including better targeting capacity. (Mitchell et al., 2021) Among the NP-based drug carriers, polysaccharides are the most experimented materials due to their unique physicochemical properties. (Liu et al., 2008) As an FDA-approved homo polysaccharide of glucose, dextran (Dex) exhibits high biodegradability, excellent biocompatibility, wide availability, low cost and easy modifiability. Thus, Dex has been used as a drug delivery vehicle for a variety of drugs. (Almeida et al., 2013; Anirudhan and Binusreejayan, 2016; Raveendran et al., 2016)

In this work, we developed ACT-conjugated dextran NPs (ACT-Dex NPs) for the controlled delivery of ACT and systemic treatment of psoriasis. The NPs were prepared by ultrasonic emulsification, followed by solvent evaporation. We showed that ACT-Dex NPs could ameliorate psoriasis-like skin disease significantly at a low dosage which does not cause side effects, while neat ACT drugs at an equivalent dosage provided much less benefit. Moreover, we demonstrated that ACT-Dex NPs suppressed keratinocyte proliferation more efficiently than neat ACT drugs by enhancing the inhibitory effect on STAT3 phosphorylation.

## 2 Material and Methods

### 2.1 Materials

ACT was obtained from Meilunbio (Dalian, China). Poly (vinyl alcohol) (PVA, hydrolyzation rate 87%–89%, 13–23 kDa), dextran (10 kDa), 1-ethyl-3-(3-(dimethylamino)propyl)-carbodiimide hydrochloride (EDC·HCl), and 4-dimethylaminopyridine (DMAP) was purchased from Sigma-Aldrich (St. Louis, MO, USA.). ACT and dextran were esterified to obtain ACT-conjugated dextran (ACT-Dex). Briefly, ACT (0.5 g) and dextran (0.5 g) were dissolved in dimethylsulfoxide (DMSO, 20 ml), followed by adding an excess amount of EDC (0.73 g) and DMAP (0.26 g). After 48 h of reaction at room temperature, the resultant ACT-Dex was precipitated in water and purified with water 3 times. The final product was a bright yellow powder after lyophilization. 1H NMR spectra were obtained from an NMR spectrometer (Bruker AV400, Billerica, MA, U.S.A.). δ (DMSO-d6, ppm): 2.06 (s, 3H), 2.08 (s, 3H), 2.18 (s, 3H), 2.25 (s, 3H), 2.29 (s, 3H), 5.79 (s, 1H), 6.24–6.33 (m, 2H), 6.4 (d, 1H), 6.69 (s, 1H), 6.73 (d, 1H), 7.02–7.10 (dd, 1H), 3.05–4.70 (m). The ACT content in ACT-Dex was determined by a standard curve method using a UV–vis spectrometer (Shimadzu 1800, Tokyo, Japan).

### 2.2 Preparation of ACT-Dex NPs

ACT-Dex NPs were prepared as reported previously. (Li et al., 2021) Briefly, a solution of ACT-Dex (50 mg) in chloroform (5 ml) was mixed with 50 ml of an aqueous solution of PVA (3 mg/ml). Subsequently, the mixture was sonicated for 5 min to obtain a uniform emulsion using a probe sonicator (settings: 25% power; (10 s)-(5 s) on-off program). After thorough evaporation of chloroform, the ACT-Dex NPs were obtained by repetitive centrifuge and redispersion. The morphology, hydrodynamic diameter (*D*_h_), and size distribution of ACT-Dex NPs were obtained using a Hitachi TM4000Plus transmission electron microscope (TEM, Tokyo, Japan, 100 kV) and a Malvern Nano-ZS90 zetasizer (Malvern, United Kingdom).

### 2.3 *In Vitro* Drug Release

The *in vitro* ACT release was determined by a dialysis method. A solution of ACT-Dex NPs in phosphate-buffered saline (PBS, 2 ml) was dialyzed against 18 ml of PBS containing 0.02% Tween 80 at 37°C. At each certain time point, 2 ml of solution were collected, and 2 ml of fresh PBS was supplied. The concentrations of released ACT were determined through UV-vis spectra.

### 2.4 Cell Culture

HaCaT cells (Human keratinocytes) were provided by China Center for Type Culture Collection and were cultured in Dulbecco’s modified Eagle medium (Gibco, Grand Island, NY, USA) supplied with 10% fetal bovine serum (Gibco) and penicillin/streptomycin (Gibco, 100 U/mL) under 100% humidity containing 5% CO_2_ at 37°C.

### 2.5 Cell Uptake of ACT-Dex NPs

To test cellular uptake of NPs, flow cytometry and confocal laser scanning microscope (CLSM) were performed using phycoerythrin (PE)-labeled ACT-Dex NPs. HaCaT cells were treated with PE-labeled ACT-Dex NPs with different concentrations for various time courses. The cells were gently washed with PBS to remove free ACT-Dex NPs. The intracellular fluorescence intensity of different concentrations (for 4 h) and different hours (with 10 μg/ml) were acquired by flow cytometry (LSR II; BD Bioscience, San Jose, USA). CLSM was performed to confirm the intracellular delivery of ACT-Dex NPs. HaCaT cells were seeded and treated with PE-labeled ACT-Dex (10 μg/ml) for 2 and 4 h individually. The cells were washed with PBS, followed by fixed with 4% paraformaldehyde (PFA). The fixed cells were stained with 4′,6-diamidino-2-phenylindole (DAPI, Beyotime, China). Intracellular fluorescence intensity and localization were observed and analyzed using CLSM (Olympus FV500, Tokyo, Japan).

### 2.6 Cell Viability Assay

Cell viability was evaluated using a Cell Counting Kit-8 assay kit (CCK-8, Beyotime, China). Briefly, HaCaT cells were incubated in a 96-well plate overnight. Cells were then treated with PBS, Dextran, or different concentrations of ACT-Dex NPs for 24 h. Next, 10 μL of CCK-8 reagent was added to each well. The absorbance at 450 nm was measured using a microplate reader (TECAN Infinite F50, Switzerland) two tours later.

### 2.7 Measurement of STAT3 Expression *In Vitro*

Western blot analysis was performed to determine STAT3 expression *in vitro*. HaCaT cells were treated with PBS, Dextran (20 μg/ml), ACT (20 μg/ml)or ACT-Dex NPs (20 µg ACT equiv./mL) for 12 h. Subsequently, the cells were further stimulated using recombinant IL-22 (PeproTech; 50 ng/ml) for an additional 1 h, and the cell lysate was prepared. After electrophoresis, proteins were electroeluted onto a polyvinylidenedifluoride (PVDF) membrane (Invitrogen, Carlsbad, USA). Antibodies: anti-pSTAT3 (Tyr705) (Cell Signaling, Beverly, USA), STAT3 (Cell Signaling), and anti-actin (Abcam, Cambridge, United Kingdom). For the visualization of the immunoreactive proteins, an enhanced chemiluminescence assay kit and a chemiluminescence imaging system (ChemiScope 6200T, ClinX, Shanghai, China) were used.

### 2.8 *In Vivo* Effect Studies of ACT-Dex NPs

#### 2.8.1 IMQ-Induced Psoriasis-like Murine Model and *In Vivo* Treatment

Wild-type Balb/c female mice (6–8 weeks) were provided by the Institute of Laboratory Animal Science (Beijing, China). The animal experiments were approved by the Animal Experimentation Ethics Committee of HUST and performed according to the protocols from the Hubei Provincial Animal Care and Use Committee. Mice received daily topical treatment of IMQ (62.5 mg of commercially available IMQ cream) on their back skin for 6 consecutive days to fabricate the psoriasis model. 2 hours after IMQ cream application, mice were treated with an intraperitoneal injection of Dex (20 μg), ACT (20 μg), ACT-Dex NPs (20 µg ACT equiv.), or PBS for 6 consecutive days.

#### 2.8.2 Psoriasis-like Lesion Evaluation

Disease severity was assessed with a clinical scoring system based on the Psoriasis Area and Severity Index (PASI) scores as previously reported.(van der Fits et al., 2009) The severity of inflammation was reflected by the cumulative score of scaling, erythema, and thickness (0–4). The back skinfold thickness was measured daily in triplicate using a vernier caliper before treatment. Data were presented as the percentage change from baseline.

#### 2.8.3 Histological and Immunohistochemical Analysis

The full-thickness skin samples on the region of interest were obtained, fixed with 4% PFA, and embedded with paraffin on day 7th once the mice were sacrificed. Then, the sections (thickness: 5 μm) of these tissues were stained with hematoxylin and eosin (H&E) and observed and photographed under a microscope. Epidermal thickness was measured using ImageJ software (National Institutes of Health, USA). Immunohistochemical studies were performed to detect the expression of Ki67 and pSTAT3 proteins on skin sections. Briefly, the sections were prepared by dewaxing and hydration, antigen retrieval, blocking, incubation with anti-ki67 antibody (1:1000; Abcam) or anti-pSTAT3 (Tyr705) antibody (1:1000; Abcam) and HRP-coupled secondary antibody (1:1000; Proteintech; China), diaminobenzidine (DAB) development, and H&E staining, respectively. Finally, the slides were observed and photographed under a microscope. Protein expression was quantified using Image J software.

#### 2.8.4 Body Weight and Spleen Weight

Body weights of the mice were recorded on day 1st and day 7th when they were sacrificed. Their spleens were then removed and weighed. Spleen index was calculated by the weight ratio between spleen and body.

#### 2.8.5 Hematological and Organ Toxicity Studies

Blood samples of all groups were obtained on day 7th before being sacrificed. Afterward, blood cell counts, aspartate aminotransferase (AST), serum levels of alanine aminotransferase (ALT), creatinine (CREA), and blood urea nitrogen (BUN) were measured following standard protocol. Major organs including livers, lungs, kidneys, and hearts were also harvested and fixed. H&E staining was performed as described above. Nikon Ni-E (Nikon, Tokyo, Japan) was used to image the H&E sections.

### 2.9 Statistical Analysis

Data were shown as means ± standard deviation unless otherwise noted. The statistical significance between different groups was analyzed by unpaired Student’s *t*-test and one-way ANOVA. Statistical significance was confirmed when *p* < 0.05.

## 3 Results and Discussion

### 3.1 Preparation and Characterization of ACT-Dex NPs

**FIGURE 1:**
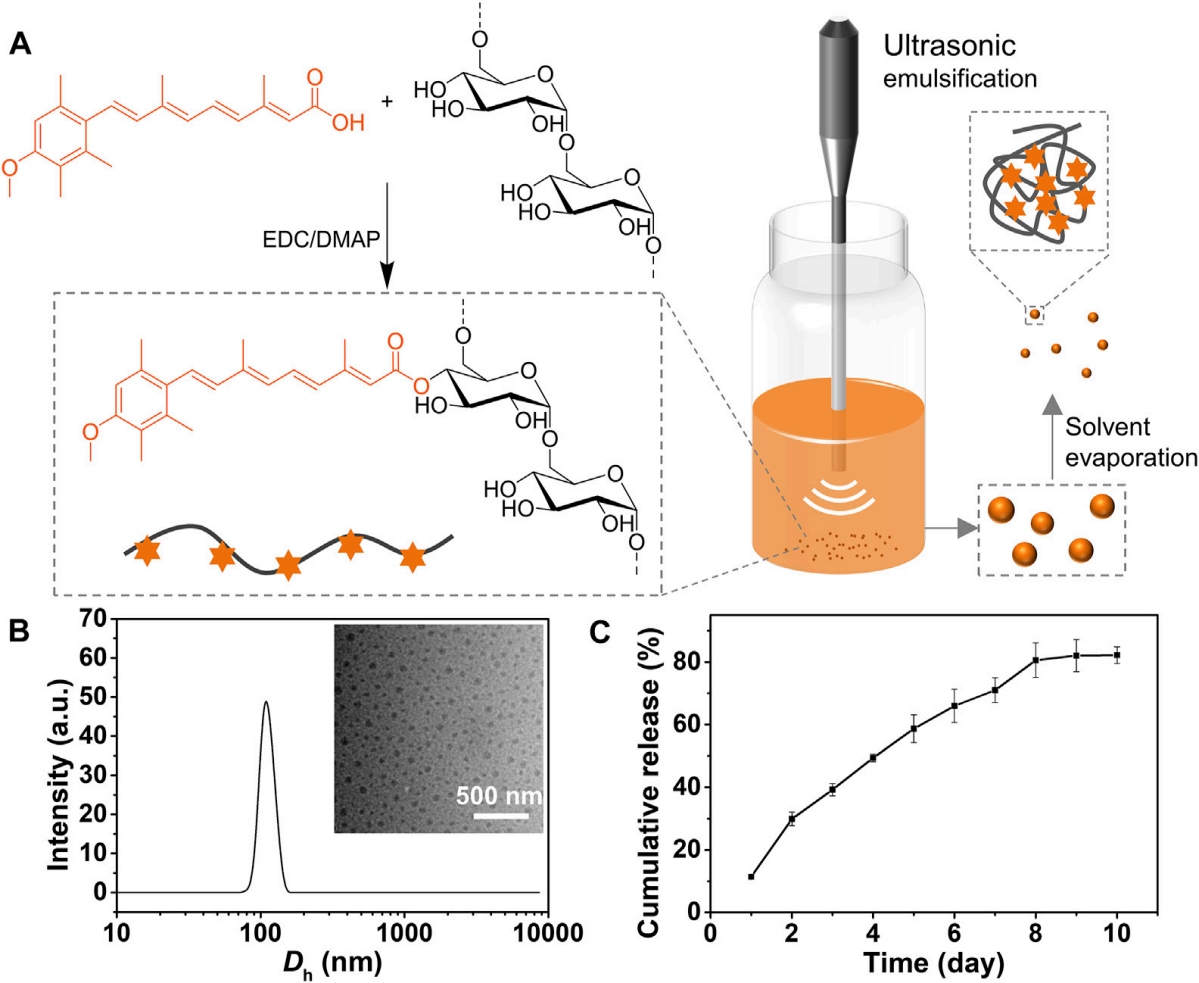
Synthesis, characterization of ACT-Dex NPs. **(A)** Schematic illustration of the synthesis of ACT-Dex conjugate and preparation of ACT-Dex NPs. **(B)** Hydrodynamic diameter (*D*_h_) and representative TEM image (inset) of ACT-Dex NPs. **(C)** *In vitro* ACT release from the NPs. Data were presented as mean ± standard deviation of three independent experiments.

The ACT-Dex conjugate was synthesized by an EDC/DMAP-catalyzed esterification between ACT and dextran (Figure 1A). 1H NMR was performed to confirm the structure of the resultant ACT-Dex (Supplementary Figure S1). All characteristic peaks were well assigned, indicating the successful conjugation of ACT to dextran. It was quite challenging to quantify the amount of ACT in this conjugate by NMR integrations because the ACT-Dex conjugate formed self-assembled structures even in organic solvents. The reason can be ascribed to the formation of hydrogen bonds and the unfavorable solubility of both dextran and ACT moieties in organic solvents after esterification. Since the UV-vis absorption of ACT at 360 nm displayed a linear relationship with the concentration (Supplementary Figure S1), UV-vis spectroscopy was employed to confirm and quantify the amount of ACT in ACT-Dex. The ACT content in ACT-Dex was calculated to be 52.9%, indicating that 1.79 repeat units of dextran on average were conjugated with one molecule of ACT.

The ACT-Dex NPs were prepared as we reported previously (Figure 1A). (Li et al., 2021) The aqueous solution of ACT-Dex NPs showed a clear and yellow color appearance. The morphology and size distribution of ACT-Dex NPs were determined by TEM and dynamic laser scattering (DLS) (Figure 1B). Clearly, the ACT-Dex NPs displayed an average hydrodynamic diameter (*D*_h_) of ∼ 100 nm with narrow size distribution. The NPs showed round-shape, well-dispersed, and no aggregation in the TEM image, indicating the good stability of the NPs.

Since the drug release behavior is usually related to the pharmacokinetics of a drug, we evaluated the drug release of ACT from ACT-Dex NPs (Figure 1C). Due to the slow hydrolysis of ester bonds, ACT was continuously released from the ACT-Dex NPs within 8 days and reached a platform thereafter, with a terminal cumulative release ratio of ∼ 80%. This sustained-release behavior may benefit the patients by reducing the administration frequency.

### 3.2 Superior Amelioration of Psoriasis-Like Skin Disease by ACT-Dex NPs

**FIGURE 2:**
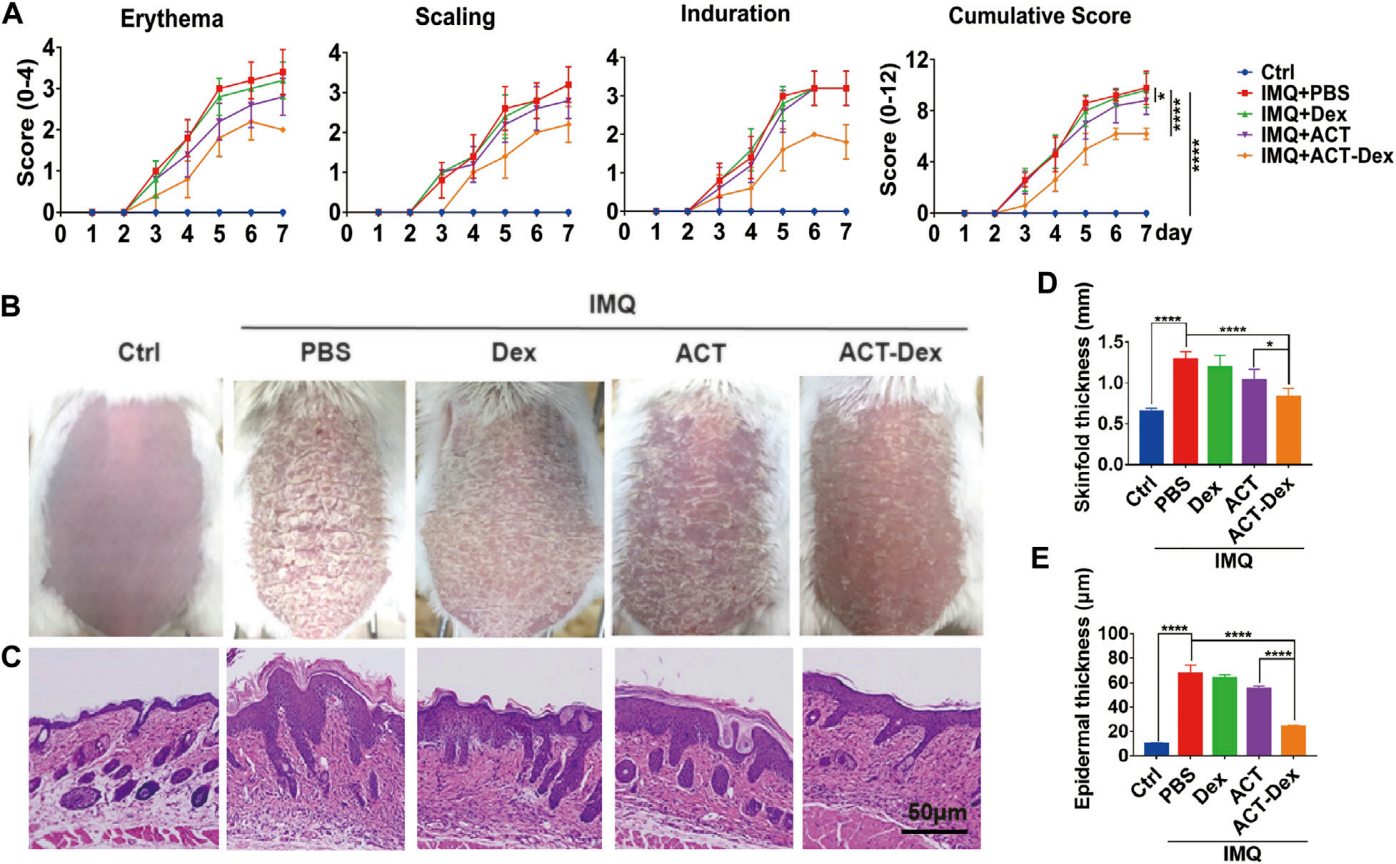
Therapeutic effects of different treatments. **(A)** Psoriasis area and severity index (PASI) scores of skin lesions in IMQ-induced psoriatic mice following different treatments. **(B)** Representative photographs and **(C)** H&E staining of the back skin derived from mice in different groups on day 7th. **(D)** Skinfold thickness and **(E)** epidermal thickness of the back skin derived from mice in different groups. Data were presented as mean ± standard deviation of three independent experiments. (*p < 0.05, ****p < 0.0001, two-tailed Student’s *t*-tests). The scale bar in the bottom right figure of **(C)** applies to the others.

We next investigated whether ACT-Dex NPs could exhibit better therapeutic effects on psoriasis-like skin disease than neat ACT drugs *in vivo*. Generally, the therapeutic effect of ACT is dose-dependent and significantly reduced at a lower dosage. A previous study showed that the psoriasis-like murine skin lesions were improved when intragastrically administrated ACT at a dosage of 168 mg/day, which is obviously a high dosage. (Pang et al., 2018) An *et al.* injected intraperitoneally a combination of low-dose ACT and methotrexate, which is at 20 µg/day, into a psoriatic murine model. (An et al., 2017) Therefore, we administrated neat ACT drugs at 20 µg/day and ACT-Dex NPs at 20 µg ACT equiv./day and compared the therapeutic efficacy of them at an equivalent low dosage. Disease severity was evaluated daily using PASI scores. We found the ACT-Dex NPs group showed a significant impact on alleviating the severity of clinical symptoms on day 3rd, including erythema, scaling, and induration, while neat ACT drugs showed a weak therapeutic effect from day 5th (Figure 2A). Consistent with PASI scores, the lesions of mice in the Dex group were similar to the PBS group. In contrast, conditions were slightly improved in the ACT group and significantly improved in the ACT-Dex NPs group (Figure 2B). The thickness of IMQ-treated skin increased to 1.296 ± 0.04 mm, while it was repressed to 1.038 ± 0.06 mm by ACT and 0.834 ± 0.04 mm by ACT-Dex NPs. The difference between the ACT group and ACT-Dex NPs group was significant (*p* < 0.05) (Figure 2C). Furthermore, the histological analyses of the back-skin lesions were performed by H&E staining. Compared to other treatment groups, treatment with ACT-Dex NPs resulted in decreased epidermal thickness and inflammatory cell infiltration, indicating the superior therapeutic effects of ACT-Dex NPs (Figure 2D). The epidermal layer of IMQ-treated skin was thickened to 67.55 ± 3.46 μm, while the epidermal thickness of ACT and ACT-Dex NPs groups were only 55.2 ± 0.93 μm and 24.21 ± 0.53 μm (*p* < 0.0001), respectively (Figure 2E).

**FIGURE 3:**
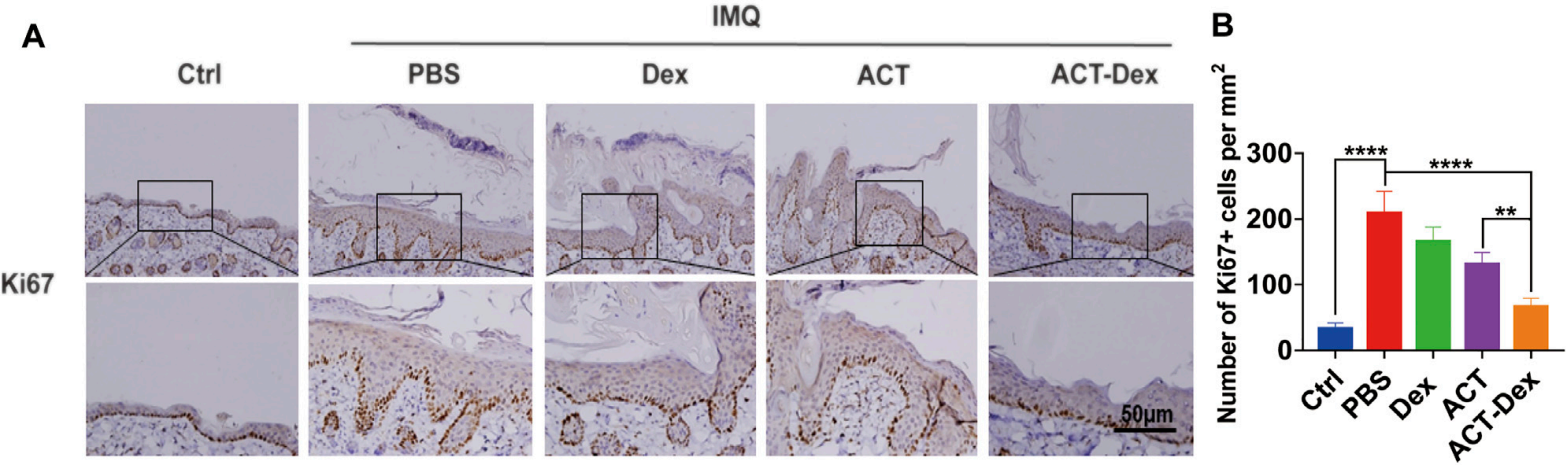
*In vivo* Ki67 expression in different treatments.**(A)** Representative Ki67 IHC staining images of the skin sections in theIMQ-induced psoriatic mice following different treatments. **(B)** The number of Ki67 + cells per square millimeter. (**p < 0.01, ****p < 0.0001, two-tailed Student’s t-tests). The scale bar in the bottom right figure of **(A)** applies to the others.

Generally, the keratinocytes are excessively proliferated in psoriatic skin lesions, and the inflammatory cascade in lesions is critically dependent on keratinocytes. (Garzorz-Stark and Eyerich, 2019; Moos et al., 2019) We thus analyzed the expression of Ki67, a marker of the proliferation of keratinocytes, in the epidermis by Ki67 IHC staining. (Ramezani et al., 2019) We found a remarkable Ki67 upregulation in the PBS and Dex groups, whereas it was slightly inhibited in the ACT group and profoundly inhibited by the ACT-Dex NPs (Figures 3A,B, Supplementary Figure S2A). These findings suggested that ACT-Dex NPs were more efficient in ameliorating psoriasis-like murine skin disease than neat ACT drugs at an equivalent low dosage.

### 3.3 Efficient Uptake of ACT-Dex NPs by Keratinocytes

**FIGURE 4:**
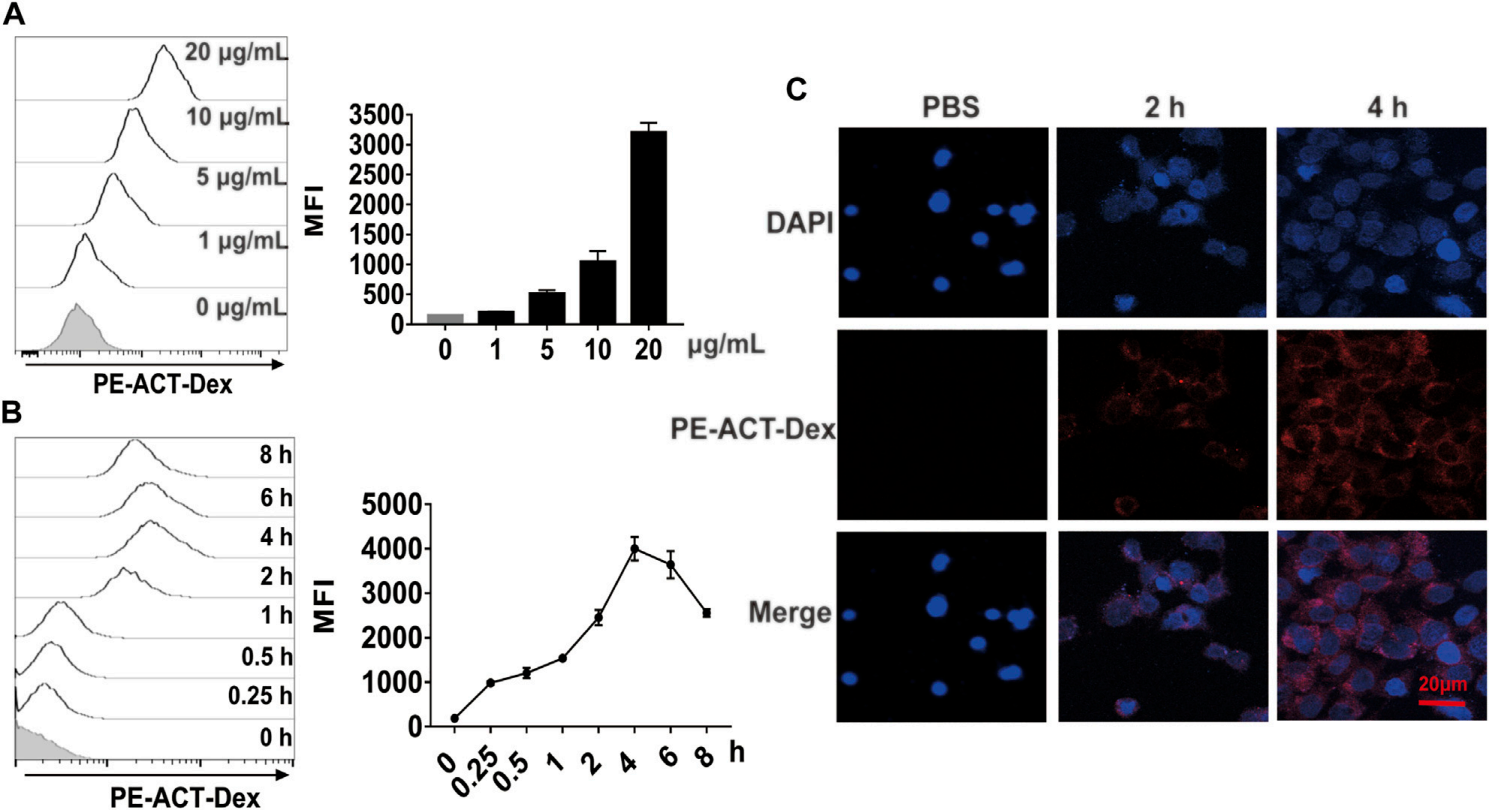
Uptake performance of ACT-Dex NPs by keratinocytes. **(A,B)** Representative flow cytometry histograms and mean fluorescence intensity (MFI) of intracellular uptake of PE-labeled ACT-Dex NPs into HaCaT cells. HaCaT cells were incubated with the indicated concentrations of PE-labeled ACT-Dex NPs for 4 h or with 10 μg/mL PE-labeled ACT-Dex NPs for the indicated times. **(C)** CLSM images of HaCaT cells internalized PE-labeled ACT-Dex NPs for various time.

The efficacy of ACT on the proliferation of keratinocytes is contingent on the internalization level of ACT by the target cells. (Pang et al., 2008; Ormerod et al., 2010) Therefore, we investigated the cellular uptake level of PE-labeled ACT-Dex NPs in HaCaT cells. HaCaT cells were incubated with different dosages of PE-labeled ACT-Dex NPs for various durations, and flow cytometry was used to analyze the intracellular fluorescence intensity. We found that there was a gradual increase in the intensity of intracellular fluorescence with the increased concentration of ACT-Dex NPs (Figure 4A) and reached the maximum at 4 h (Figure 4B). CLSM also indicated similar results (Figure 4C). These findings suggested that ACT-Dex NPs could be internalized into keratinocytes efficiently.

### 3.4 Better Suppression Effects of ACT-Dex NPs on Psoriatic Keratinocyte Proliferation by Enhanced Inhibition of STAT3 Phosphorylation

ACT could ameliorate psoriasis *via* suppressing keratinocyte proliferation, while the molecular mechanism of it remains unclear. Previous studies indicated that ACT could bind with intracellular retinoic acid-binding protein (CRABP) to translocate to nucleus, then formed RAR and RXR heterodimer complexes which could regulate the expression of over 500 genes after binding to the gene promoter region. (Balmer and Blomhoff, 2002; Larange and Cheroutre, 2016)

STAT3, a member of the STAT family, is vital for cell survival, proliferation, differentiation, and immune responses. (Levy and Darnell, 2002; Yu et al., 2007; Miklossy et al., 2013) Previous studies showed that the constitutive activation of STAT3 was observed in the epidermis of psoriatic skin lesions. (Sano et al., 2005) Also, the activation of STAT3 in keratinocytes in a murine model could develop a skin phenotype closely resembling psoriasis. (Miyoshi et al., 2011; Calautti et al., 2018) These results indicated that activated STAT3 in keratinocytes played a critical role in psoriasis development. Accordingly, we explored the influence of ACT-Dex NPs on STAT3 in comparison with neat ACT.

**FIGURE 5:**
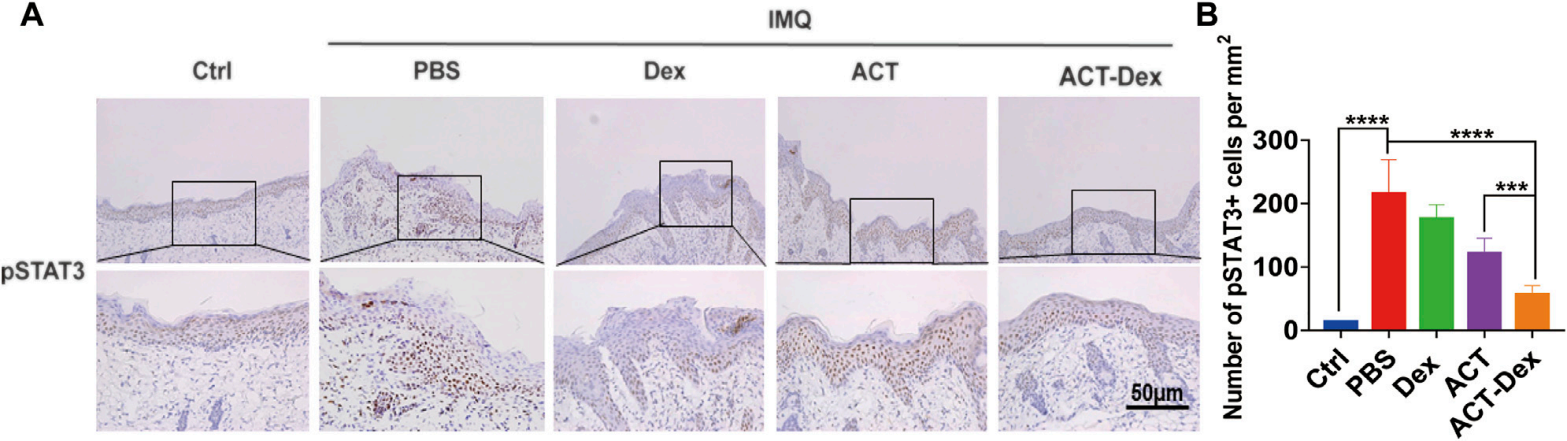
*In vivo* pSTAT3 expression in different treatments. **(A)** Representative pSTAT3 IHC staining images of the skin sections in the imiquimod-induced psoriatic mouse model from different groups. **(B)** The number of pSTAT3+ cells per square millimeter. pSTAT3, phosphorylated STAT3. (***p < 0.001, ****p < 0.0001, two-tailed Student’s t-tests). The scale bar in the bottom right figure of **(A)** applies to the others.

We first analyzed the skin lesions of psoriasis-like murine models treated by different drugs by phosphorylated STAT3 (pSTAT3) IHC staining. As expected, the expressions of pSTAT3 in the epidermis were strongly enhanced in PBS and Dex groups, while it was slightly inhibited in the ACT group and significantly inhibited in the ACT-Dex NPs group (Figures 5A,B, Supplementary Figure S2B)**.**

**FIGURE 6:**
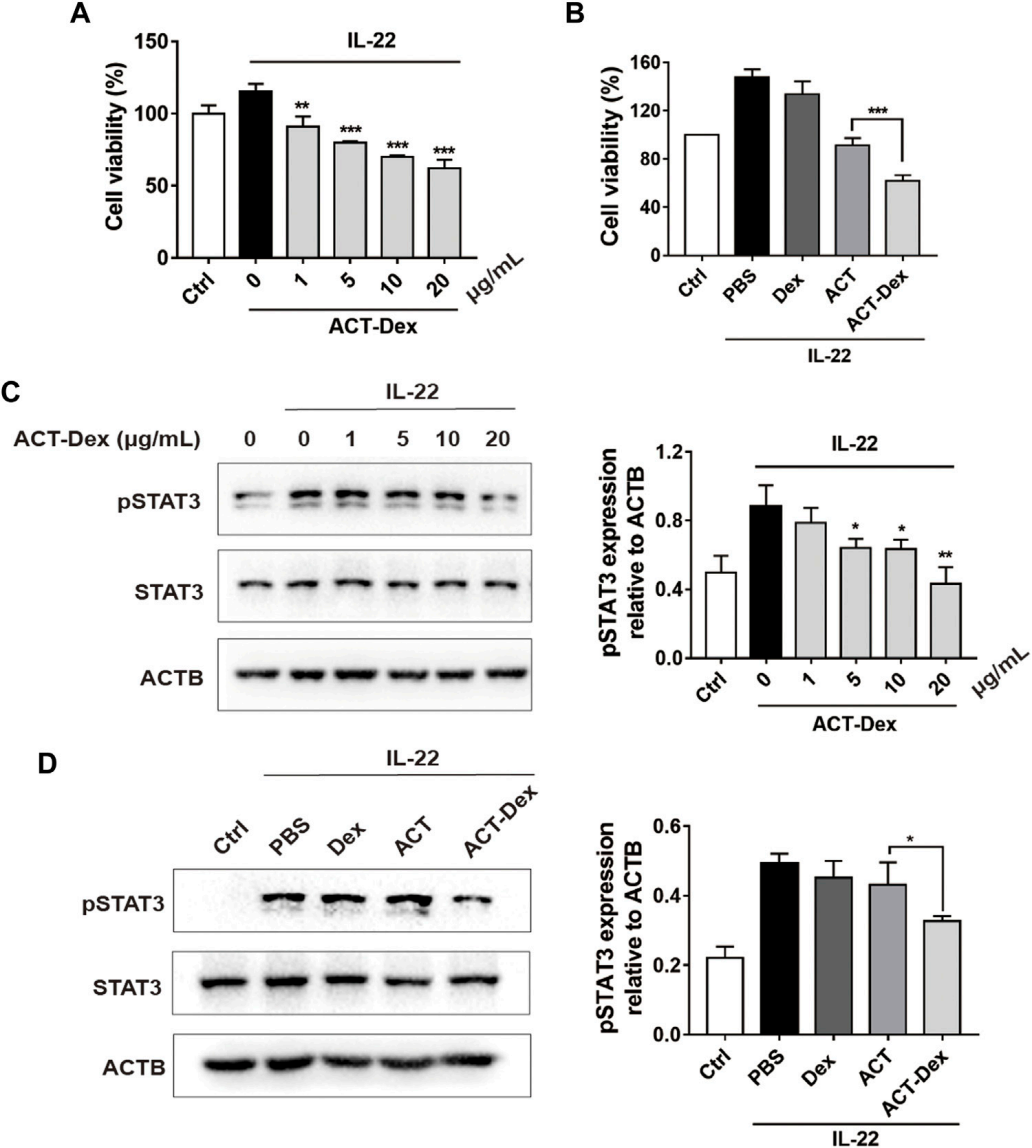
Inhibitory effects of ACT-Dex NPs on keratinocytes. **(A)** Viability of IL-22-stimulated HaCaT cells by treating with ACT-Dex NPs at the indicated concentrations and **(B)** by treating with PBS, Dex, ACT, or ACT-Dex NPs. **(C,D)** Western blot images and densitometric measurements of the expression level of pSTAT3 in IL-22-stimulated HaCaT cells by treating with ACT-Dex NPs (20 μg/ml) at the indicated concentrations and by treating with PBS, Dex (20 μg/ml), ACT (20 μg/ml), or ACT-Dex NPs (20 µg ACT equiv./mL) for 12 h pSTAT3, phosphorylated STAT3. Data are means ± standard deviation of three independent experiments. (**p < 0.01, and ***p < 0.001, two-tailed Student’s *t*-tests).

We next conducted *in vitro* experiments to explore the influence of ACT-Dex NPs on the proliferation of STAT3-activated keratinocytes and compare it to neat ACT drugs. Considering IL-22 plays an important role in the pathogenesis of psoriasis and is a major activator of STAT3 signaling. We thus stimulated HaCaT cells with IL-22 to simulate the abnormal keratinocytes in psoriasis, followed by treating with ACT-Dex NPs. (Sestito et al., 2011; Johnston and Gudjonsson, 2014; Nikamo et al., 2014; Tohyama et al., 2018) Cell proliferation detected by CCK8 assay showed that ACT-Dex NPs caused a dose-dependent inhibition of proliferation in IL-22-stimulated HaCaT cells (Figure 6A). We next compared the suppressing effects on HaCaT cells by ACT-Dex NPs and neat ACT drugs. IL-22-stimulated HaCaT cells were treated with PBS, Dex (20 μg/ml), ACT (20 μg/ml) and ACT-Dex NPs (20 µg ACT equiv./mL). Surprisingly, we found that ACT-Dex NPs inhibited the proliferation of IL-22-stimulated HaCaT cells by 58.22%, and the inhibitory effect was significantly stronger than that of neat ACT drugs (38.23%) (Figure 6B). Correspondingly, ACT-Dex NPs treatment caused a dose-dependent inhibition of STAT3 phosphorylation in IL-22-stimulated HaCaT cells, as shown by western blot analysis (Figure 6C). And activating phosphorylation of STAT3 was blocked by ACT-Dex NPs more significantly than neat ACT drugs (Figure 6D). Therefore, these results suggested that ACT-Dex NPs ameliorate psoriasis-like skin disease more efficiently than neat ACT by an enhanced inhibitory effect on STAT3 phosphorylation of psoriatic keratinocytes.

### 3.5 Systemic Impacts and Safety of ACT-Dex NPs

The systemic impacts and safety of ACT-Dex NPs were evaluated. At the end of 7-days treatments, the body weights of any IMQ-treated groups remained above 90% of original values and no significant difference among these groups was observed (Supplementary Figure S3A). The increase in the spleen/body weight ratio could be caused by the increase of cell population in the spleen. (Cesta, 2006) As expected, the spleen/body weight ratio significantly increased in IMQ treated mice, but there was no significant decrease in other treatment groups. This indicated that the influence of low-dose ACT on the systemic immune system was not obvious (Supplementary Figures S3B,C).

**FIGURE 7:**
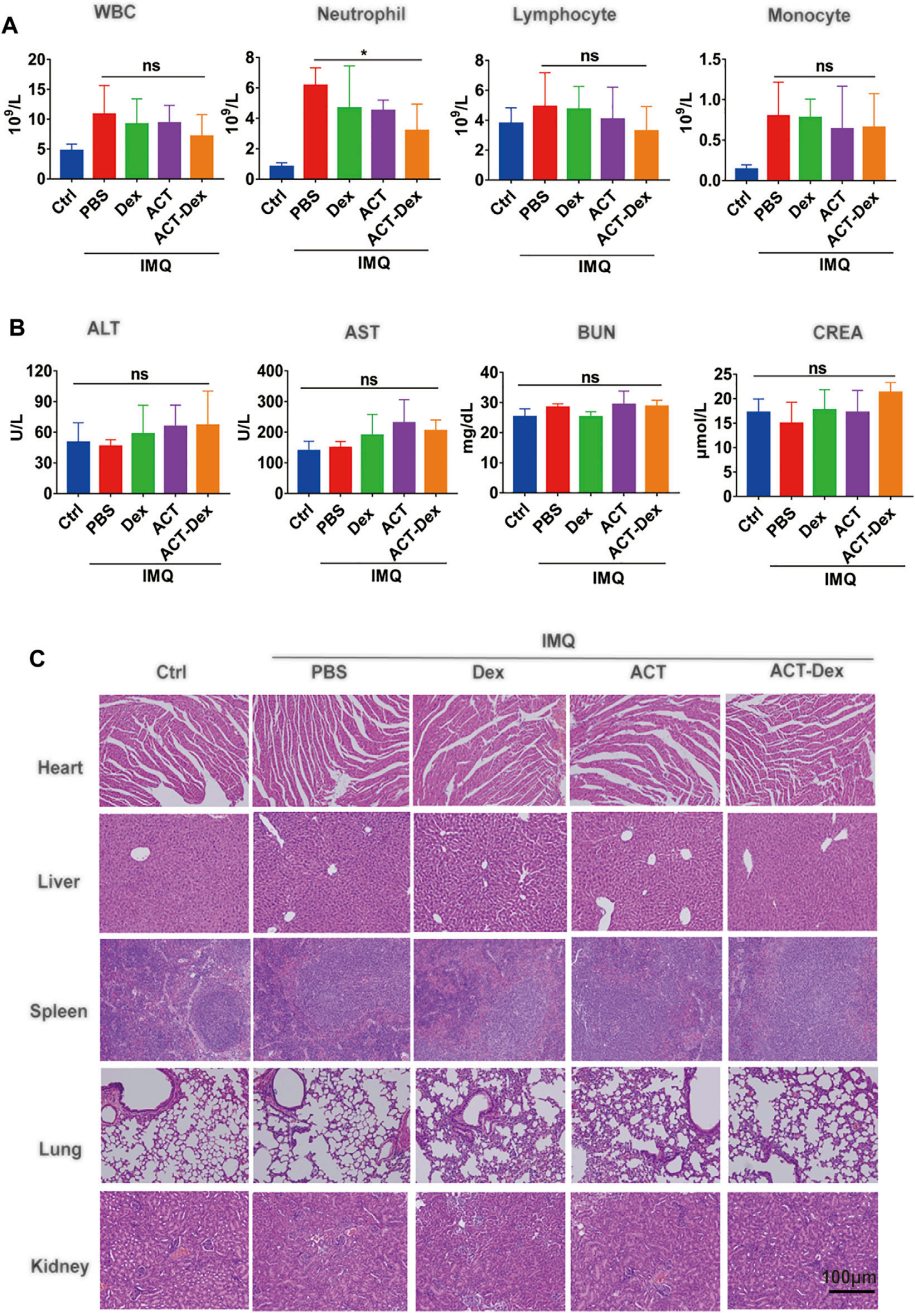
Biocompatibility of ACT-Dex NPs in mice. **(A)** Blood cell counts and **(B)** blood biochemistry of mice in different groups at day 7th. **(C)** Representative images of H&E staining of the heart, lung, liver, spleen and kidney derived from mice in different groups at day 7th. The scale bar in the last image of **(C)** applies to the others. (ns: not significance; *p < 0.05; two-tailed Student’s *t*-tests.

The counts of white blood cells (WBC), neutrophils, monocytes, and lymphocytes were evaluated. No significant differences between all groups were observed, suggesting that the administration of 20.0 μg ACT or ACT-Dex NPs did not affect the hematopoietic system of the psoriatic mice (Figure 7A). Besides, negligible chage in the serum levels of ALT, AST, CREA, and BUN in all groups was observed, indicating that no acute liver or renal toxicities were induced at the dosage of this study (Figure 7B). As the H&E staining of major organs, no detectable tissue damage was observed in all groups (Figure 7C), suggesting that the administration of 20.0 μg ACT or ACT-Dex NPs did not cause histopathological abnormities. The above results indicate the superior biocompatibility and safety of ACT-Dex NPs for *in vivo* psoriasis treatment.

## 4 Conclusion

In summary, ACT-Dex NPs were developed and proved to ameliorate psoriasis more effectively than neat ACT drugs at an equivalent low dosage. The low-dose (1 mg/kg/day) administration did not cause any adverse systemic events. Moreover, we demonstrated that ACT-Dex NPs inhibited keratinocyte proliferation more efficiently than neat ACT drugs by enhancing the inhibitory effect on STAT3 phosphorylation. This study indicated that ACT-Dex NPs could be a new candidate for psoriasis with higher efficacy and safety.

## Data Availability

The original contributions presented in the study are included in the article/Supplementary Material, further inquiries can be directed to the corresponding authors.
