# Supplementary material for: Acitretin-Conjugated Dextran Nanoparticles Ameliorate Psoriasis-like Skin Disease at Low Dosages
Source: Front Bioeng Biotechnol. 2022 Jan 7;9:816757. doi: 10.3389/fbioe.2021.816757 (PMC8777251; doi:10.3389/fbioe.2021.816757)
Supplement: Supplementary file 1 [file DataSheet2.docx]

Supplementary Material

# Supplementary Figures

##
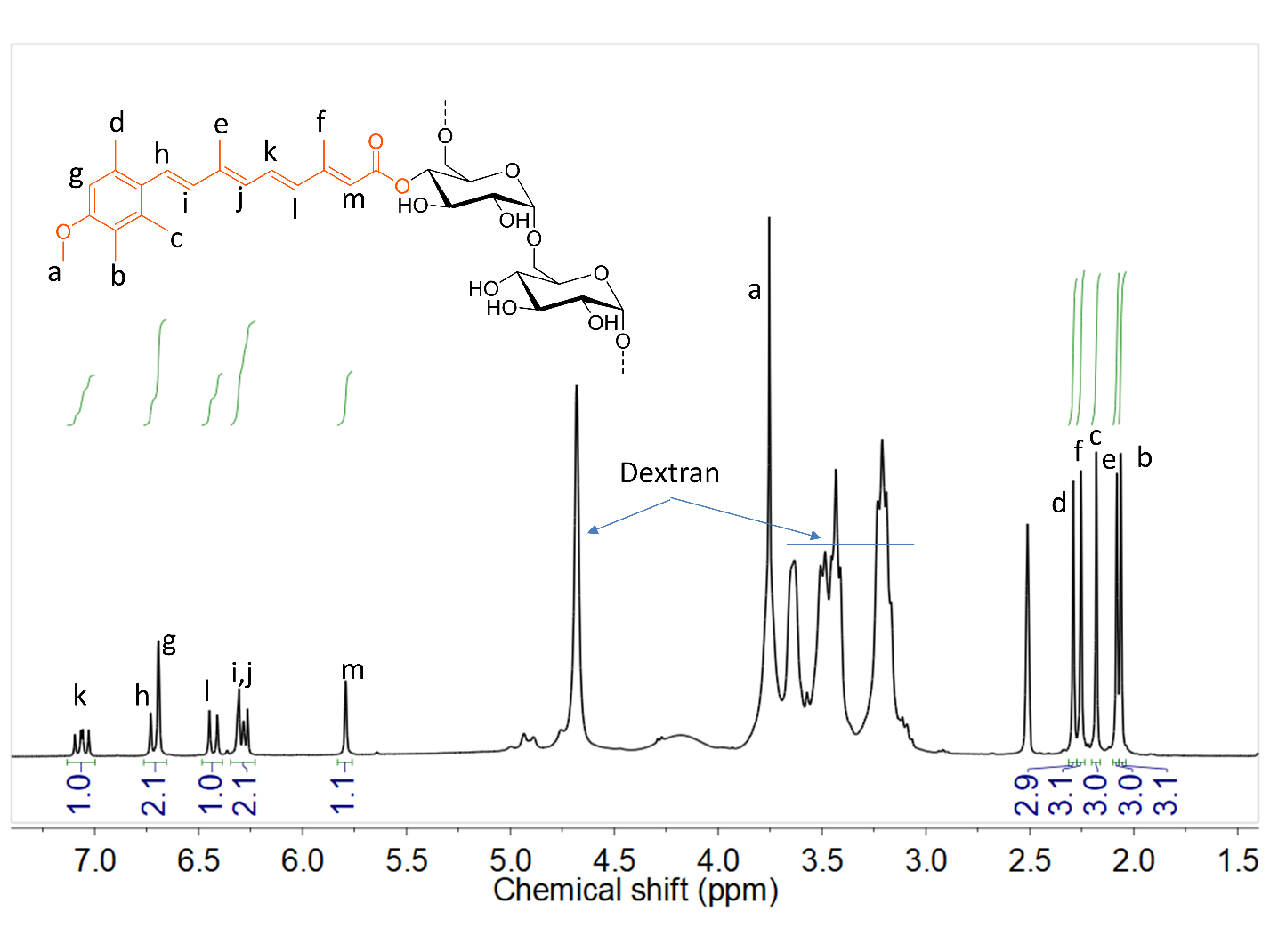
Supplementary Figure 1. ^1^H NMR spectrum of ACT-Dex in DMSO-*d*6.


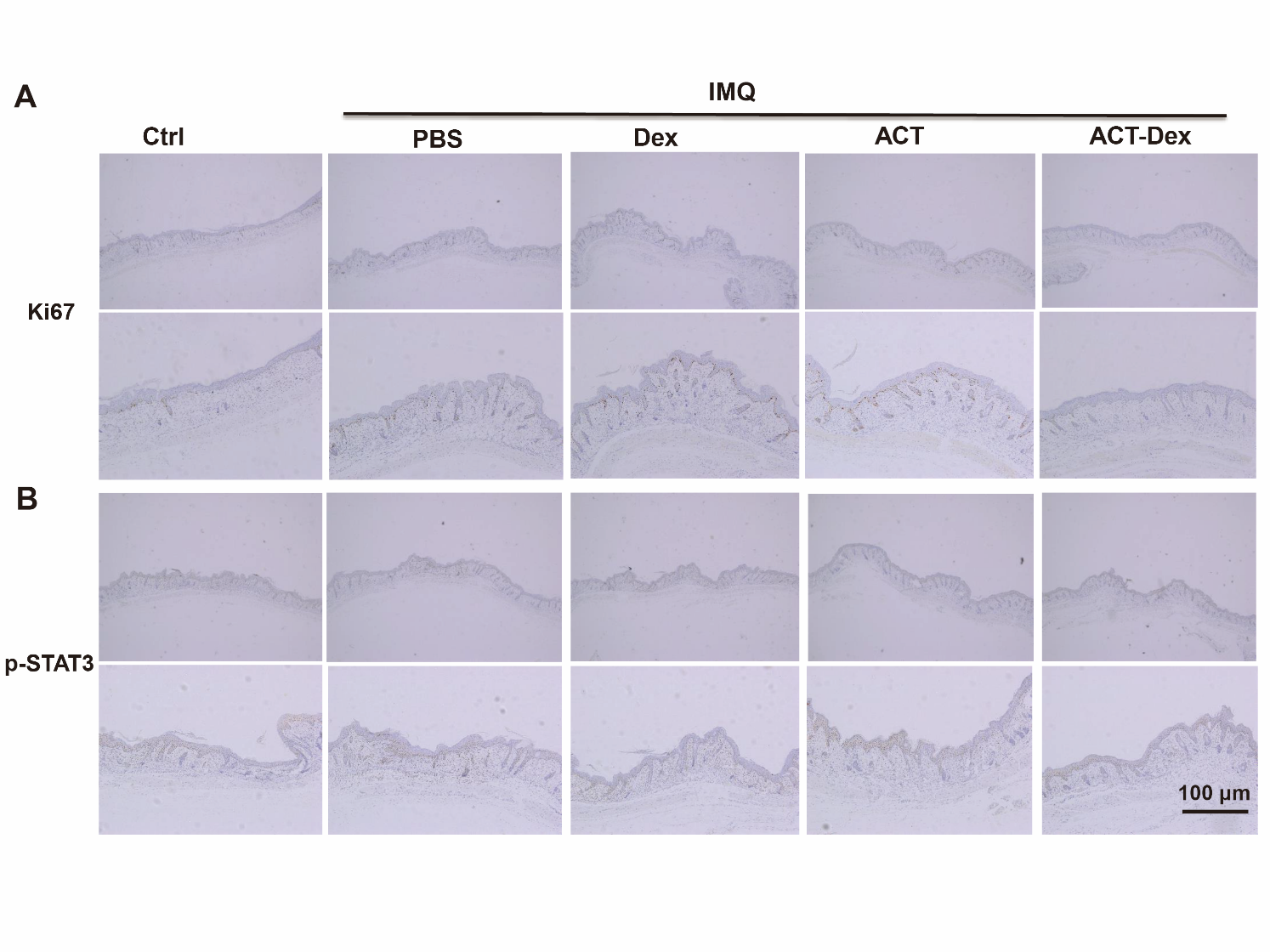


**Supplementary Figure 2.** Representative IHC staining images of (A) Ki67 and (B) p-STAT3 of the skin sections in the IMQ-induced psoriatic mice following different treatments. The scale bar in the bottom right figure of (B) applies to the others.

**
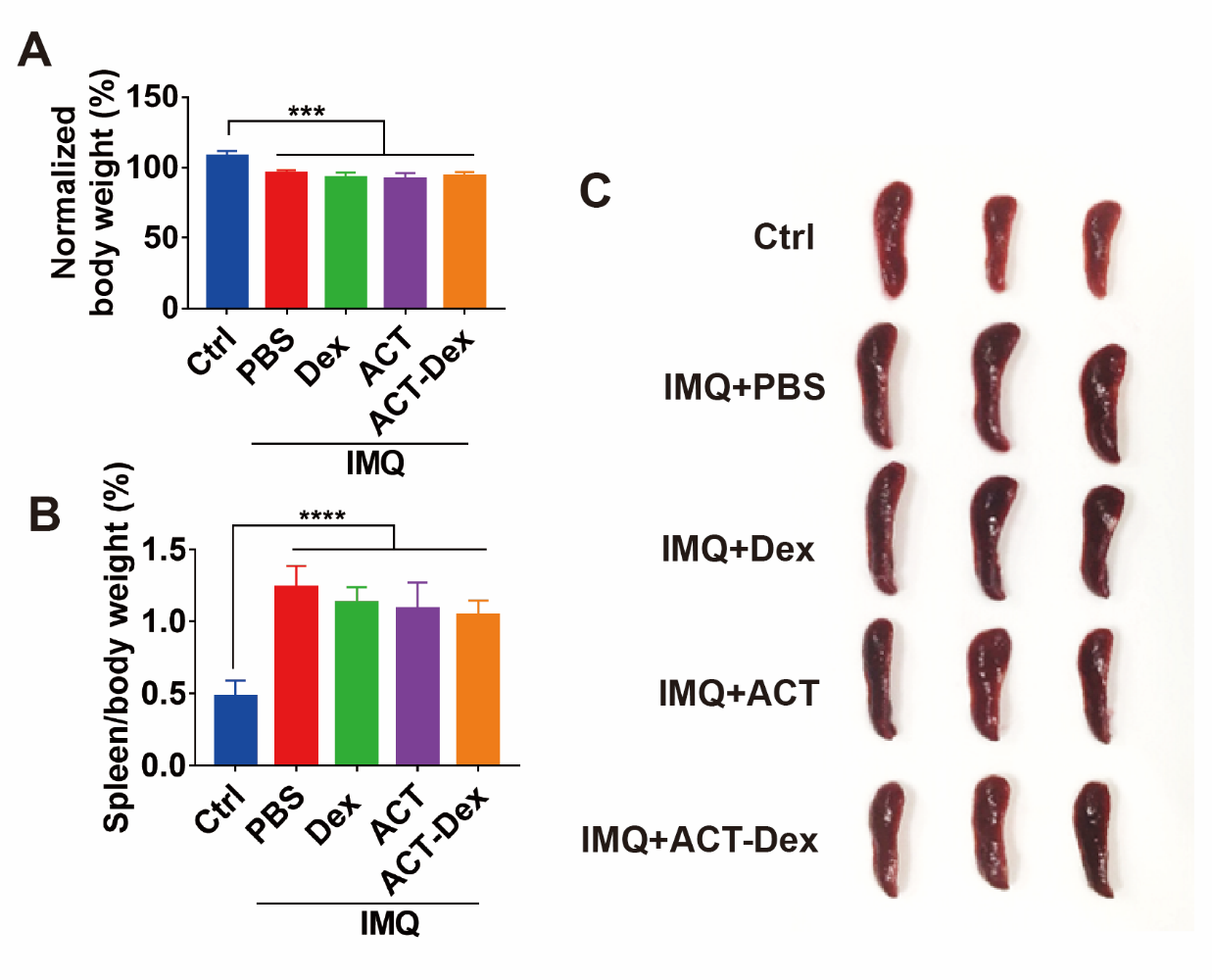
**

## Supplementary Figure 3. Body and spleen weight changes in different groups. (A) Body weight on day 7^th^ compared to that on day 0. (B) Representative gross image of spleens at day 7^th^. (C) Spleen/body weight ratios of mice in different groups on day 7^th^. (****p* < 0.001, *****p* < 0.0001; two-tailed Student’s *t*-tests).
