# Supplementary material for: Acitretin-Conjugated Dextran Nanoparticles Ameliorate Psoriasis-like Skin Disease at Low Dosages
Source: Front Bioeng Biotechnol. 2022 Jan 7;9:816757. doi: 10.3389/fbioe.2021.816757 (PMC8777251; doi:10.3389/fbioe.2021.816757)
Supplement: Supplementary file 2 [file DataSheet1.docx]

For the Table of Contents Use Only:

Title: Acitretin-Conjugated Dextran Nanoparticles Ameliorate Psoriasis-like Skin Disease at Low Dosages

**Author:** Jiajia Lan, Yuce Li, Jingjing Wen, Yu Chen, Jing Yang, Liang Zhao, Yuting Xia, Hongyao Du, Juan Tao, Yan Li*, Jintao Zhu*

**Graph:**


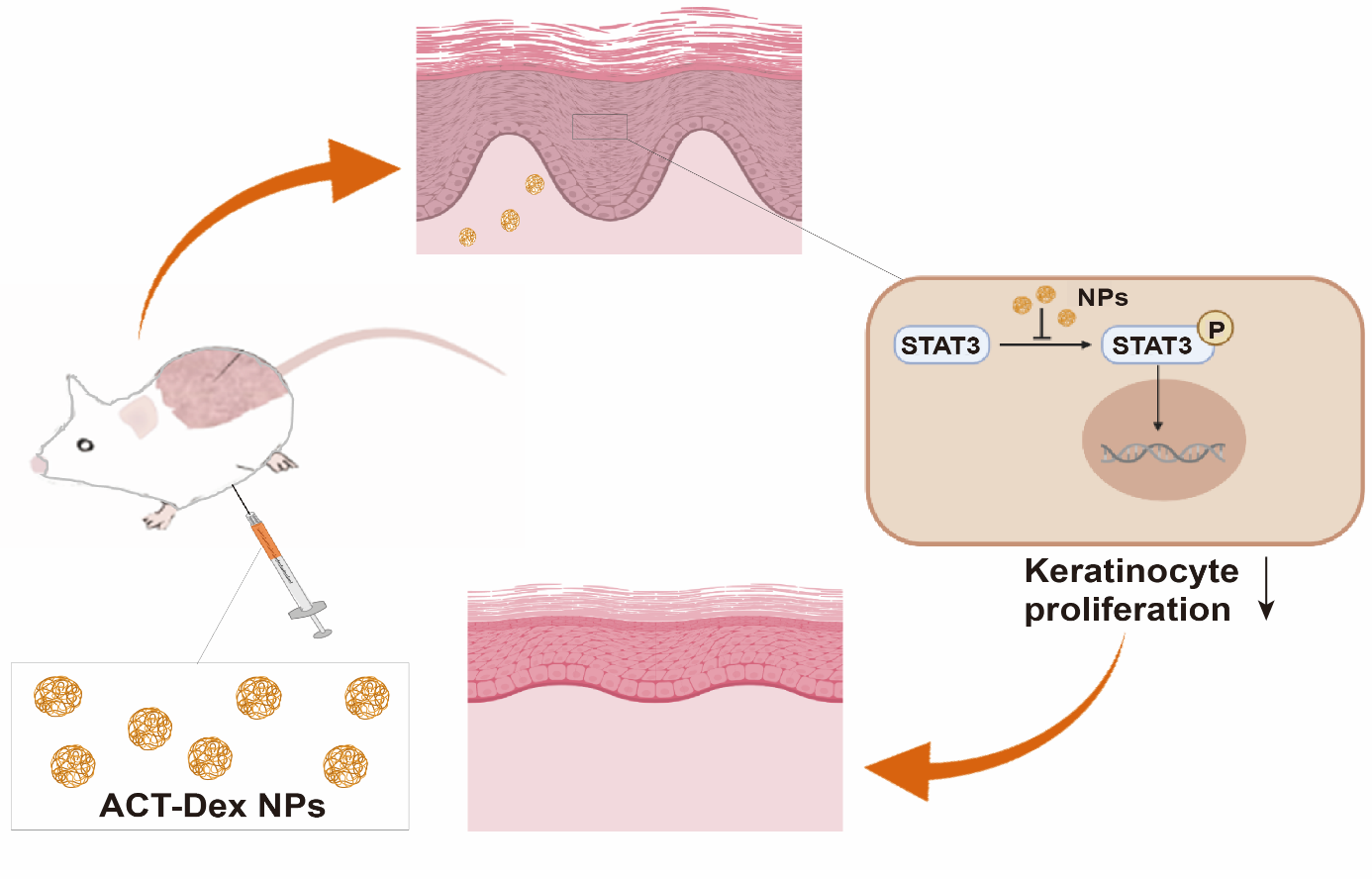


**ToC text:**

Acitretin-conjugated dextran nanoparticles (ACT-Dex NPs) are developed to improve the therapeutic efficacy of ACT on psoriasis. ACT-Dex NPs could ameliorate psoriasis-like skin disease more significantly than neat ACT drugs at an equivalent low dosage. The NPs suppress keratinocyte proliferation more efficiently by enhancing the inhibition of STAT3 phosphorylation. ACT-Dex NPs provide new opportunities for effective and safe treatment of psoriasis.
